# Endometriosis Imaging Diagnosis Beyond Specialized Centers: A Retrospective Cross-Sectional Study

**DOI:** 10.7759/cureus.99050

**Published:** 2025-12-12

**Authors:** Soha Wahab, Zeina Chehade, Miguel Luna Russo, Miran A Jaffa

**Affiliations:** 1 Faculty of Medicine, American University of Beirut Medical Center, Beirut, LBN; 2 Obstetrics and Gynecology, University of Texas at San Antonio, San Antonio, USA; 3 Obstetrics and Gynecology, American University of Beirut Medical Center, Beirut, LBN; 4 Obstetrics and Gynecology, Institute for Women’s Health and Body, Complex Gynecology and Surgery, West Palm Beach, USA; 5 Epidemiology and Population Health, American University of Beirut, Beirut, LBN

**Keywords:** deep endometriosis, deep infiltrating endometriosis (die), endometriosis pain, endometriosis surgery, ovarian endometrioma, ovarian endometriosis, women's imaging endometriosis

## Abstract

Objective: Determine the accuracy of deep endometriosis (DE) and ovarian endometrioma (OE) diagnosis on pelvic MRI in non-specialized tertiary healthcare, taking surgical diagnosis as the reference test. Additionally, we aimed to evaluate symptom severity and the presence and size of endometriomas as predictors of DE detection during surgery.

Methods: This retrospective cross-sectional study assessed endometriosis patients presenting to a tertiary healthcare center over one year (2018-2019). Pelvic MRI data, obtained within six months of surgery, were extracted from radiologic reports. A symptom severity score (SSS) was used to estimate symptom severity.

Results: Pelvic MRI exhibited low sensitivity (75.6%) and high specificity (100%) for DE (n=96). Conversely, OE was diagnosed with high sensitivity (100%) and specificity (93.9%; n=122). Three-way contingency analysis showed that, irrespective of the presence and size of endometrioma, there was a correlation between symptom severity and detection of DE during surgery (OR=6.6, p-value≤0.001; n=287). In a multivariable logistic regression analysis model controlling for endometrioma size, a higher SSS was significantly associated with a higher risk of DE detection during surgery (SSS=1: OR=4.65, p≤0.001; SSS=2: OR=10.66, p≤0.001; n=287). However, when controlling for symptom severity, there was no significant association between the presence and size of endometrioma and surgical detection of DE (endometrioma ≥5 cm: OR=1.57, p>0.05; n=287).

Conclusion: While pelvic MRI effectively identifies DE in this population, caution is advised regarding negative DE MRI results in non-specialized settings. Based on our findings, a high suspicion for DE should be maintained in patients with severe symptoms.

## Introduction

Endometriosis is a prevalent disease, affecting up to 15% of females, with an average delay in diagnosis of eight to 12 years [1]. It results from the presence of endometrial-like tissue in extrauterine sites that can be categorized as ovarian endometrioma (OE), superficial endometriosis, or deep endometriosis (DE), the latter traditionally defined as endometriosis that infiltrates surrounding structures by more than 5 mm [2,3]. Both DE and endometrioma may be diagnosed with non-invasive imaging, yet laparoscopy remains the gold-standard diagnostic tool for endometriosis, specifically superficial lesions [4]. In its latest guidelines, the European Society of Human Reproduction and Embryology (ESHRE) recommends considering endometriosis diagnosis in patients exhibiting cyclical or non-cyclical symptoms suggestive of the disease. ESHRE also advises further investigation with non-invasive diagnostic imaging (MRI or ultrasound), even when the clinical examination appears normal [5]. The value of non-invasive diagnostic imaging extends beyond its safety and cost-effectiveness; it also lies in its usefulness for pre-operative planning and assessing the need for a multidisciplinary surgical team to ensure adequate surgical management. When transvaginal ultrasound (TVUS) includes the assessment of DE in multiple locations, such as the uterosacral ligaments and rectum, the diagnostic accuracy for DE detection becomes similar and even superior to the surgical diagnosis considered the gold standard [6]. In light of these findings, the Society of Radiologists in Ultrasound convened a panel of experts and released a consensus on recommendations and techniques to improve DE diagnosis using TVUS [7]. For pelvic MRI, significant variability persists in the protocols used and the accuracy reported in the literature [8]. Consequently, the European Society of Urogenital Radiology (ESUR) developed recommendations to enhance detection and characterization of pelvic endometriosis using MRI, particularly DE [8]. However, in clinical practice, standardized protocols for endometriosis diagnosis via non-invasive imaging remain underused despite their potential to improve diagnostic accuracy, and this point is often overlooked in endometriosis-related guidelines [9].

In this cross-sectional study, our primary objective is to evaluate the accuracy of endometrioma and DE diagnosis using MRI scans performed at a tertiary healthcare center that lacks specialized endometriosis services, with surgical findings serving as the reference test. This center represents many similar institutions worldwide commonly accessed by patients with endometriosis. We also aim to evaluate predictors of DE diagnosis during surgery, particularly focusing on the presence of severe symptoms and endometriomas. The true prevalence of DE remains unclear, underscoring the importance of such investigations in clinical practice [10,11].

This article was previously presented as an abstract poster at the AAGL 53rd Global Congress on November 16, 2024.

## Materials and methods

This was a retrospective cross-sectional study employing convenience sampling conducted at the American University of Beirut Medical Center (AUBMC), Beirut, Lebanon.

Eligibility criteria

All patients aged 18 or older who presented to AUBMC between 2018 and 2019 and had ICD (International Classification of Diseases)-10 codes consistent with suspected or confirmed endometriosis were included.

To ensure that the study population predominantly consisted of patients accurately diagnosed with endometriosis involving the pelvic or abdominal cavity, the following categories of patients were excluded: 1 - patients with isolated abdominal wall endometriosis related to a previous C-section and without evidence of pelvic cavity involvement, and 2 - patients initially suspected of having endometriosis who were subsequently found to have no evidence of disease on both imaging (MRI and ultrasound) and surgical evaluation.

Quality measures

Data collection and analysis were conducted following approval by the center’s Institutional Review Board of the American University of Beirut Medical Center (BIO-2021-0372, dated January 17, 2022) and in accordance with ethical research guidelines. Clinical data were extracted from the EPIC electronic medical record (EMR) system, which was implemented in 2018. All data were de-identified before analysis, and no patients were contacted as part of this study. One author verified the collected data by cross-referencing EMR records, and discrepancies were resolved by consensus.

Data collection

Pelvic ultrasounds were performed either by a gynecologist in an outpatient clinic or by a technician in the Radiology Department, with findings recorded in the EPIC database. Pelvic MRIs were conducted using 1.5 T or 3 T machines, following a protocol implemented for benign gynecologic conditions at the AUBMC Radiology Department. MRI images included T2 weights (axial, sagittal, coronal, and axial fat suppressed) and T1 weights (axial and coronal fat suppressed before and after gadolinium administration). Radiologists interpreting MRI images had completed a five-year radiology degree, with or without additional fellowship training unrelated to pelvic MRI.

In evaluating the accuracy of pelvic MRI, we considered sub-populations who underwent MRI within six months of surgery and whose operative reports clearly stated the presence or absence of DE and endometrioma, respectively.

The presence of DE during surgery, considered the reference test, was assessed using specific criteria in the operative report and notes, which included partial or complete obliteration of the cul-de-sac, presence of a frozen pelvis, diagnosis of stage 3 or 4 disease as per the American Society for Reproductive Medicine (ASRM) endometriosis classification system, identification of severe endometriosis, or description of a distinct DE lesion such as a fibrotic lesion within the layers of the bowel.

A symptom severity score (SSS) ranging from 0 to 3 was employed to estimate the severity of symptoms, specifically focusing on pain symptoms that significantly impact patients' daily lives, including chronic pelvic pain (CPP), dyspareunia, and neuropathic pain. The absence of an alternative validated scoring system drove the decision to use this non-validated score. CPP is defined as the presence of pain in the pelvis outside menstruation for at least six months [12]. Neuropathic pain is pain caused by a lesion or disease of the somatosensory system [13]. Symptoms of neuropathic pain may vary, described in our population as burning or shooting pain often radiating to the legs or groin.

Statistical analysis

The diagnostic accuracy of pelvic MRI for detecting DE and endometrioma was assessed using surgical findings as the reference standard. Sensitivity, specificity, false-positive and false-negative rates, and Cohen’s kappa coefficient were calculated to evaluate MRI performance and agreement. Associations between SSS, endometrioma size, and the presence of DE during surgery were analyzed using three-way contingency analysis. Multivariable logistic regression models were then performed to identify independent predictors of DE, incorporating endometrioma size and symptom severity categories, with odds ratios and 95% CIs reported. Statistical significance was defined as p≤0.05, and all analyses were conducted using STATA version 17 (StataCorp LLC, College Station, TX).

## Results

Table 1 delineates the findings of the non-invasive diagnostic imaging, namely pelvic MRI and pelvic ultrasound: DE, endometrioma, DE and endometrioma, and no endometriosis on imaging. Pelvic ultrasound was performed on all 688 patients, whereas pelvic MRI was done on approximately half of them (n=356, 51.7%). Notably, DE was never visualized during pelvic ultrasound, whether performed abdominally or vaginally. On pelvic MRI, DE was identified either as a well-defined nodule or more broadly as “thickening of the uterosacral ligaments suggestive of DE.” Isolated endometrioma on imaging was the most prevalent finding (n=383, 55.7%), but only 31.6% of these patients underwent a pelvic MRI, which may detect DE. Moreover, 155 (22.5%) patients were found to have both DE and endometrioma, 67 (9.7%) patients had isolated DE, and 83 (12.1%) patients had negative imaging for endometriosis, of whom 17 (20.5%) were diagnosed with endometriosis based on clinical symptoms without surgical confirmation.

**Table 1:** Non-invasive imaging diagnosis of endometriosis in tertiary healthcare (N=688 patients) ^a^DE was diagnosed only on MRI, not on TVUS DE, deep endometriosis; TVUS, transvaginal ultrasound

Variable	Category	n (%)
Pelvic imaging performed	Pelvic abdominal ultrasound or TVUS	688 (100)
	Pelvic MRI	356 (51.7)
Endometriosis seen on imaging	DE^a^	67 (9.7)
	Endometrioma	383 (55.7)
	DE and endometrioma	155 (22.5)
	No endometriosis on imaging	83 (12.1)

Table 2 and Table 3 summarize the diagnostic accuracy of MRI in detecting DE and endometrioma, respectively, among patients whose surgical reports clearly documented the presence or absence of DE and OE. The tables include sensitivity, specificity, false-positive and false-negative rates, and kappa coefficients, with surgery used as the reference standard. In Table 2, pelvic MRI demonstrated low sensitivity (75.6%) but high specificity (100%) for DE (n=96), with a kappa coefficient of 0.39, indicating poor agreement between MRI and surgical diagnosis in this non-specialized tertiary setting. In contrast, Table 3 shows a remarkably high level of agreement between endometrioma diagnosis on MRI and the corresponding surgical diagnosis (kappa=0.96), with high sensitivity (100%) and specificity (93.9%; n=122).

**Table 2:** Accuracy of DE diagnosis on pelvic MRI in non-specialized tertiary healthcare (N=96 patients) Included cases (N=96): cases who underwent pelvic MRI within six months of surgery, and the judgment for the presence of DE during surgery was clear as “yes” or “no” based on the operative report and pathological specimens. Significance: p-value<0.05 DE, deep endometriosis

DE diagnosis	DE diagnosis in surgery		
DE diagnosis on MRI prior to surgery	DE diagnosis result	Positive	Negative
	Positive	65	0
	Negative	21	10
Sensitivity (%)	75.6		
Specificity (%)	100		
False positive (%)	0		
False negative (%)	24.4		
Kappa	0.39 (p≤0.001)		

**Table 3:** Accuracy of endometrioma diagnosis on pelvic MRI in non-specialized tertiary healthcare (N=122 patients) Included cases (N=122): Cases who underwent pelvic MRI within six months of surgery and for whom the judgment of the presence of endometrioma during surgery was clearly recorded as "yes" or "no" based on the operative report and pathological specimens. Significance: p-value<0.05

Endometrioma diagnosis	Endometrioma diagnosis in surgery		
Endometrioma diagnosis on MRI prior to surgery	Endometrioma diagnosis result	Positive	Negative
	Positive	89	2
	Negative	0	31
Sensitivity (%)	100		
Specificity (%)	93.9		
False positive (%)	6.1		
False negative (%)	0		
Kappa	0.96 (p≤0.001)		

The status of DE during surgery was unclear in 146 records, either due to insufficient postoperative documentation with a lack of histopathological specimens or due to inaccessible surgical findings for procedures done outside AUBMC. Table 4 presents the three-way contingency analysis based on the Mantel-Haenszel odds ratio (MH OR) to examine the correlation between endometriomas >2 cm, symptom severity, and DE status during surgery. Endometrioma size was assessed through imaging modalities (ultrasound or MRI). For the 146 records with uncertain DE status, we assumed negative findings, given the belief that DE presents in a minority of endometriosis cases [12]. The analysis demonstrated an independent significant association between symptom severity and DE status during surgery. Specifically, patients with severe symptoms had 3.55 times the odds of having DE compared to those with less severe symptoms (MH OR=3.55, 95% CI (2.36-5.34), p<0.001).

**Table 4:** Association between the presence of endometrioma >2 cm, severe symptoms, and DE status during surgery (N=433 patients) ^a^SSS = CPP + dyspareunia + neuropathic pain; range (0-3) Included cases (N=433): patients who underwent surgery for endometriosis. Significance: p-value<0.05 DE, deep endometriosis; MHOR, Mantel-Haenszel odds ratio; SSA, symptoms severity score; CPP, chronic pelvic pain

Presence of endometrioma >2 cm in surgery	SSS^a^	DE present in surgery	DE absent in surgery/cannot tell
Endometrioma >2 cm present (N=308)	0	56	78
	1	60	40
	2	50	12
	3	11	1
Endometrioma >2 cm absent (N=125)	0	11	30
	1	20	16
	2	28	11
	3	6	3
MH OR	3.55 (p≤0.001)		

Additionally, we performed the same analysis considering only patients with clear surgical DE status, and the corresponding results are included in Table 5. This analysis showed an even stronger association between symptom severity and surgical diagnosis of DE, with an odds ratio of 6.6 (MH OR=6.6, 95% CI (3.26-13.4), p<0.001).

**Table 5:** Association between presence of endometrioma >2 cm, severe symptoms, and presence or absence of DE during surgery (N=287 patients) ^a^SSS = CPP + dyspareunia + neuropathic pain; range (0-3) Included cases (N=287): patients who underwent surgery for endometriosis, and the judgment for the presence of DE during surgery was clear as “yes” or “no” based on the operative report and/or pathological specimens. Significance: p-value<0.05 DE, deep endometriosis; MHOR, Mantel-Haenszel odds ratio; SSA, symptoms severity score; CPP, chronic pelvic pain

Presence of endometrioma >2 cm	SSS^a^	DE present in surgery	DE absent in surgery
Endometrioma >2 cm present (N=207)	0	56	21
	1	60	6
	2	50	3
	3	11	0
Endometrioma >2 cm absent (N=80)	0	11	11
	1	20	3
	2	28	1
	3	6	0
MH OR	6.6 (p≤0.001)		

To further confirm our findings, we performed a multivariable logistic regression analysis to determine the adjusted association between SSS values, the presence and size of the endometrioma, and DE status during surgery. As in the MH OR analysis, we conducted the analysis twice: first assuming DE was absent in the 146 records with uncertain DE status (Table 6), and then considering only patients with clear DE status (Table 7). A noticeable trend emerged in both Table 6 and Table 7, showing a progressively stronger and statistically significant association between the number of severe symptoms and surgical diagnosis of DE when controlling for the presence and size of the endometrioma. Notably, in Table 7, which only considers patients with clear surgical DE status, no odds ratio could be computed for patients presenting with all three severe symptoms (SSS=3) due to the absence of cases without DE in this category. Interestingly, neither the presence nor the size of the endometrioma demonstrated a significant association with DE during surgery in either table when controlling for symptom severity, as indicated by the 95% CI spanning across 1.

**Table 6:** Association of DE status during surgery with both the severity of symptoms and the presence or size of endometrioma using multivariable logistic regression analysis (N=433 patients) ^a^Status of DE during surgery: DE present, DE absent, cannot tell ^b^SSS = CPP + dyspareunia + neuropathic pain; range (0-3) Included cases (N=433): patients who underwent surgery for endometriosis. Significance: p-value<0.05 DE, deep endometriosis; MHOR, Mantel-Haenszel odds ratio; SSA, symptoms severity score; CPP, chronic pelvic pain

Status of DE in surgery^a^	P-value	Odds ratio	95% CI
Endometrioma size: ≤2 cm; <5 cm	0.056	1.63	0.98-2.70
Endometrioma ≥5 cm	0.05	1.69	0.99-2.89
SSS^b^=0 (reference)	NA	NA	NA
SSS=1	<0.001	2.35	1.48-3.74
SSS=2	<0.001	5.99	3.39-10.60
SSS=3	<0.001	7.75	2.46-24.36

**Table 7:** Association of presence or absence of DE during surgery with both severity of symptoms and presence or size of endometrioma using multivariable logistic regression analysis (N=287 patients) ^a^DE judged as either present or absent during surgery ^b^SSS = CPP + dyspareunia + neuropathic pain; range (0-3) Included cases (N=287): Patients who underwent surgery for endometriosis and for whom the judgment for the presence of DE during surgery was clearly stated as “yes” or “no” based on the operative report and/or pathological specimens. Significance: p-value<0.05 DE, deep endometriosis; SSA, symptoms severity score; CPP, chronic pelvic pain

Presence or absence of DE during surgery^a^	P-value	Odds ratio	95% CI
Endometrioma size: ≤2 cm; <5 cm	0.067	2.21	0.94-5.16
Endometrioma ≥5 cm	0.305	1.57	0.66-3.75
SSS^b^=0 (reference)	NA	NA	NA
SSS=1	<0.001	4.65	2.03-10.61
SSS=2	<0.001	10.66	3.52-32.33
SSS=3	NA (null values in Table 5)		

Finally, to address the clinical belief associating endometrioma size with symptom severity, we used the ordinal trend test based on the gamma statistic. The test showed a small but significant inverse relationship between endometrioma size and symptom severity (SSS) (gamma statistic=-0.1, p=0.04), challenging the hypothesis that endometriomas are the source of pain.

## Discussion

As previously outlined, all patients underwent ultrasound (either abdominal or transvaginal) due to its widespread availability in outpatient gynecology clinics. However, this diagnostic modality was limited to describing the reproductive organs and identifying free fluid in the cul-de-sac, without mentioning the sliding sign in radiological reports, which involves applying gentle pressure on the cervix to determine the mobility of the cervix and uterus over the rectum [6]. An online survey conducted among gynecologists in Greece who perform TVUS revealed that nearly all participants believed TVUS should be the primary investigative tool for patients with endometriosis. However, over 90% of respondents expressed the need for additional specialized training to diagnose DE, and more than half indicated they felt unable to diagnose bowel DE using ultrasound [14]. A study at the Mayo Clinic tracked the learning curve of an experienced radiologist who underwent intensive observational training in performing advanced TVUS for endometriosis diagnosis over one week at an expert center. The study found that proficiency in performing this examination could be achieved with fewer than 100 scans, yielding high sensitivity and specificity [15].

On the other hand, the significance of MRI lies in its ability to evaluate disease severity, particularly when there is suspicion of extra-pelvic involvement and intestinal lesions. This imaging modality facilitates optimal surgical planning, ensuring the best possible patient outcome while minimizing the need for repeat incomplete procedures [16]. However, the accuracy of DE detection on pelvic MRI varies across studies, depending on the implemented protocol, the location of the lesions, and the expertise of the reader [17]. Structured reporting of MRI results describing DE per compartment has been proposed to ensure complete documentation of findings and satisfaction of gynecological surgeons. This approach has shown comparable diagnostic performance to free-text reports and, in some cases, superior accuracy, particularly when performed by an expert radiologist with specific interest and expertise in endometriosis [17,18]. In our database, MRI reports were free-text and frequently lacked clear characterization of DE. Yet, despite the permissive description of DE on pelvic MRI as thickening of uterosacral ligaments suggestive of DE in many cases, almost one in four patients with surgically confirmed DE received a false negative result on preoperative MRI reports, while all patients with a positive MRI result had surgically confirmed DE. This underscores the inadequacy of relying solely on negative MRI results, particularly in non-specialized settings, to rule out DE. Conversely, the diagnosis of endometrioma on pelvic MRI exhibited high sensitivity and specificity, affirming its reliability as a valuable tool for both confirming and excluding the presence of this entity.

Expert utilization of non-invasive imaging for DE diagnosis can be even more sensitive than surgical evaluation alone. Goncalves et al. demonstrated that TVUS performed by an experienced sonographer is significantly more sensitive than systemic evaluation of the pelvic cavity performed by an experienced surgeon when detecting rectosigmoid and bladder endometriosis [19]. This demonstrates the pivotal role of non-invasive imaging in delineating the extent and depth of lesions, which cannot be fully appreciated during surgical evaluation alone.

In clinical practice, it is believed that severe symptoms and endometriomas are associated with DE. In a study involving 300 patients with endometrioma larger than 2 cm, Chapron et al. found that 40.3% of patients had DE excised during surgery. They also demonstrated that the mean score of each pain symptom was significantly higher in patients with DE, irrespective of lesion location. Furthermore, the intensity of the pain did not correlate with the size of the endometrioma [20]. Similar findings were reported by Rocha et al. in a large-scale retrospective study, further supporting the association between symptom severity and the presence as well as the multifocality of DE [21]. In our database, the Visual Analog Scale score for each pain symptom was unavailable. Therefore, we estimated symptom severity using SSS as an index encompassing the presence of CPP, dyspareunia, and neuropathic pain, and we controlled for the presence and size of endometrioma. Remarkably, both MH OR analysis and multivariable logistic regression analysis indicated that the presence of more severe symptoms is associated with a higher likelihood of diagnosing DE during surgery. This association remained significant regardless of the presence or size of endometrioma, which showed no significant effect. In fact, the ordinal trend test revealed a small inverse relationship between symptom severity and endometrioma size. This finding aligns with those reported by Chapron et al., suggesting that pain intensity does not positively correlate with endometrioma size [20]. These results are significant as they challenge the common trend of focusing on endometrioma as the primary source of pain in non-specialized settings. This tendency leads to overlooking the role of DE, which appears to be underdiagnosed on non-invasive imaging in such settings, as indicated by our findings.

Strengths and limitations of the study

Our study examines outcomes related to endometriosis diagnosis at a non-specialized tertiary healthcare center in the context of the most recent endometriosis guidelines, thereby shedding light on the challenges endometriosis patients encounter during diagnosis. Although clear criteria for defining a specialized endometriosis center are still lacking, the presence of a combination of factors can reliably indicate the absence of specialization. These factors include the absence of specialized imaging for diagnosing and mapping DE, the lack of multidisciplinary surgical excision for severe endometriosis, the prevalent use of ablation and incomplete excision, and the lack of clinical specialization among surgeons who manage both obstetric and gynecological cases. Such centers, which represent the majority of healthcare facilities accessed by endometriosis patients, particularly in the initial stages, would naturally be classified as non-specialized centers.

However, multiple limitations should be acknowledged. This is a retrospective study that relies on free-text input from healthcare providers in the EPIC EMR system. VAS scores (patient rating of pain severity from 1 to 10) for endometriosis symptoms were not documented. We derived a variable, SSS, to estimate severity, and the values of SSS are reproducible. Additionally, the surgical diagnosis of DE was inferred from physicians' descriptions of surgical findings, with or without histopathological confirmation. DE was excised and sent to pathology in only a minority of cases. Therefore, relying solely on pathological results to confirm surgical DE would likely miss many cases. The criteria for identifying DE based on operative reports are detailed in the Methods section. Furthermore, there was no clear description of the compartments involved with DE on MRI or operative reports in most cases. Consequently, our analysis was limited to the presence of DE without further delineation of its anatomical extent. These limitations underscore the necessity for enforced standardization in reporting endometriosis findings in both imaging and surgical contexts. Such standardization would not only improve clinical practice but also facilitate further research in this field.

## Conclusions

Non-invasive diagnostic imaging plays a crucial role in reducing diagnostic delays and facilitating pre-operative planning for optimal outcomes in endometriosis excision surgeries. However, given the absence of formally integrated advanced standardized training in imaging for endometriosis, the significant disparity between guidelines and the current state of imaging available for most endometriosis patients should be recognized in result interpretation. This is exemplified by the underdiagnosis of DE and the subsequent exaggerated emphasis on the role of endometrioma outside expert centers, especially in patients with severe symptoms. Hence, there is a need to unite individual and organizational efforts to close this gap, striving for a highly accurate diagnosis of DE through widely accessible non-invasive imaging services.
